# Effects of dominance and prestige based social status on competition for attentional resources

**DOI:** 10.1038/s41598-019-39223-0

**Published:** 2019-02-21

**Authors:** Ashton Roberts, Romina Palermo, Troy A. W. Visser

**Affiliations:** 1 School of Psychological Science, The University of Western Australia, Crawley, Western Australia Australia; 2 ARC Centre of Excellence in Cognition and its Disorders (CCD), Crawley, Western Australia Australia

## Abstract

Social status can be attained through either dominance (coercion and intimidation) or prestige (skill and respect). Individuals high in either of these status pathways are known to more readily attract gaze and covert spatial attention compared to their low-status counterparts. However it is not known if social status biases allocation of attentional resources to competing stimuli. To address this issue, we used an attentional blink paradigm to explore non-spatial attentional biases in response to face stimuli varying in dominance and prestige. Results from a series of studies consistently indicated that participants were biased towards allocating attention to low- relative to high- dominance faces. We also observed no effects of manipulating prestige on attentional bias. We attribute our results to the workings of comparatively early processing stages, separate from those mediating spatial attention shifts, which are tuned to physical features associated with low dominance. These findings challenge our current understanding of the impact of social status on attentional competition.

## Introduction

Humans often rank peers hierarchically based on a wide array of dimensions and constructs^1^. One of the most important dimensions is “social status” – a construct based on the perceived levels of certain traits or ‘valued dimensions’ such as physical strength, salary, or academic achievement^2–6^. Social status hierarchies are ubiquitous across cultures^7^, age groups^8^, and even many animal species^9,10^. Moreover, social status has a variety of behavioural consequences. For example, low status individuals will often defer decisions to their higher status counterparts and in turn benefit from social learning^4,11,12^, while high status individuals benefit from deference from others, preferential resource distribution, and perceived competence^3,13–15^.

Henrich and Gil-White’s^4^ dual model of social hierarchy suggests that status can be attained through either dominance or prestige based pathways. Dominance based status is characterised by either social dominance (i.e. control over resources or outcomes) or the use of fear to attain status, produced through intimidation, manipulation, and coercion^13,16–21^. Dominance based status is also conceptually separable from power and aggression^22^. According to the dual model, dominance is an evolution-based early pathway for status attainment, which exists in nonhuman species^23^, for which the antecedents are reflected in readily perceivable physical indicators such as a masculinised and matured face (e.g. squarer jaw)^24–26^.

Conversely, prestige based status is attained through skill, knowledge, respect, and success^11,17^. Prestige based status emerged comparatively later in evolution, and it is based on more abstract and less physically-discernable dimensions^4,22,27^. Importantly, studies have found strong evidence that social learning is a strong mediating factor between prestige and attention^4,11,12,28,29^. When people are required to learn a new skill, they will generally defer their attention and copy the actions of individuals highest in prestige. To illustrate, a study by Chudek *et al*.^29^ showed that children, when asked to choose between two types of food or drink, would copy the choice of a model that was higher in prestige (in this case – a model who received greater bystander attention in a previously viewed cued clip). This effect of prestige bias on social learning was also seen in adults when learning a novel skill^28^.

Consistent with the dual model of social hierarchy, manipulations of either dominance or prestige, and sometimes both, have been shown to affect individuals’ abilities to exert social influence^22,30–32^. For example, in one study individuals completed a collaborative group task, and both dominance and prestige predicted group influence and creations of social rank^22^. In the second part of this study, in which the eye movements of naïve participants viewing videos of group interactions in the initial study phase were examined, participants looked more frequently at individuals rated high in either dominance or prestige than individuals rated low in either dominance or prestige.

Collectively, the behavioural and social effects shown by Cheng *et al*.^22^ suggest dominance and prestige may have the ability to bias social attention (i.e. cognitive and perceptual systems dedicated to analysing the attentional focus of other individuals^33^ – see Mattan *et al*.^27^ for a review). Additional evidence for this suggestion comes from studies looking at gaze cuing, in which peripheral targets aligned with the direction of gaze of a centrally presented face are responded to faster and more accurately than non-aligned targets^34,35^, even when gaze does not predict target location. Past studies have shown that more dominant faces^36,37^ and faces of individuals with greater prestige^2^ both enhance gaze cueing. For example, Jones *et al*.^36^ presented masculinised (high dominance) and feminised (low dominance) male and female face cues for varying durations prior to the onset of peripheral targets. They found that participants responded significantly faster to targets congruent with the gaze of masculinised faces compared to targets congruent with the feminised faces at shorter cue durations, irrespective of the gender of the participant. Similarly, Dalmaso *et al*.^2^ manipulated prestige by pairing images of faces used as cues in a subsequent gaze-cueing task with fictional curricula vitae depicting persons associated with high (“graduated with honours in physics”) or low (“retired factory worker who did not complete primary school”) academic achievement. Participants consistently responded faster to targets in the direction of gaze of high achieving faces, whereas the direction of gaze of low achieving faces had no influence on performance.

In sum, there is solid evidence that social status (whether dominance or prestige based) can bias allocation of covert attention in observers^2,36,37^ as well as their eye gaze^22^. However, these studies only examine the effects of social status on reflexive and covert attention. They do not examine any effects of competition for attentional resources. As such, it is not known whether high status stimuli are also more likely than low status stimuli to be prioritised for attentional processing in the face of competing cognitive demands. It is also not known whether such a bias would apply equally to stimuli varying in dominance or prestige based status. These questions are the focus of the present study. To be clear, we are concerned here with the issue of how non-spatial attention is linked to the prestige and dominance based pathways to social status as described by the dual model of social hierarchy. This can be distinguished from the question of how attention might be influenced by outcomes such as social rank and power achieved as a result of having high social status (for a review of this literature, see Cheng and Tracy)^17^.

One method of examining these questions is using a dual-task paradigm known as the attentional blink (AB). In the conventional AB paradigm, participants are presented with two targets embedded amongst a rapid serial visual presentation (RSVP) stream^38^ of non-target distractors. Items in the RSVP are presented at a central fixated location at a rate of approximately 10 Hz, with each item masking the preceding stimulus. Participants are required to ignore all distractors, and to detect and/or identify the two targets and report them at their leisure following the last item in the RSVP. Under these conditions, participants are typically quite accurate at identifying the first target (T1), but demonstrate significantly reduced performance for the second target (T2) when it follows closely after the first – most prominently at intervals of 300–400 ms – an effect known as the attentional blink^39–42^.

A number of theories have been advanced to explain the AB (see^43^ for a review). Bottleneck theories, for example, ascribe the AB to a lack of available processing resources for T2 arising from the need to process T1^44,45^. On the other hand, both early^46^ and more recent theories attribute the AB to a competition for attentional resources between T1 and T2 (and under some conditions, temporally adjacent distractors)^47–49^. This reasoning suggests that the magnitude of the AB can be treated as an index of the ability of T2 to attract attentional resources in the face of competing attentional demands from oncoming stimuli, with a larger AB indicating less ability to attract resources.

A number of studies indicate that varying T2 properties can substantially moderate AB magnitude. For example, increasing the temporal predictability of T2 greatly reduces the AB^50–53^. The AB is also reduced when the second target is motivationally or emotionally salient^54–56^, a participants’ own name^57^, or an angry face compared to a neutral face^54,58,59^. With this work in mind, here we examined whether social status can also influence the magnitude of the AB as a means of determining whether status biases attentional allocation. To begin, in Experiment 1, we congruently and simultaneously varied the dominance and prestige associated with a T2 face in order to maximise the strength of the status manipulation. Dominance was manipulated by using male faces with that differed on pre-rated dominance. Prestige was varied in a similar manner to that of Dalmaso *et al*.^2^ by associating target faces in the AB task with CVs depicting varying levels of academic achievement. Based on previous findings that indicate covert attention and eye gaze is biased towards high status stimuli^2,27,36,37^, we hypothesised that non-spatial attention would also be significantly biased towards high- rather than low- social status stimuli at the nadir of the AB.

## Experiment 1a

### Method

#### Participants

Twenty-nine undergraduate students (*M* = 19.24, *SD* = 2.49, male = 9, female = 20) were recruited from an introductory psychology unit at the University of Western Australia. All participants reported normal or corrected-to-normal vision, and received partial course credit for their involvement.

#### Ethical approval and informed consent

This study was approved under the University of Western Australia Human Research Ethics Board (RA/4/1/8640). The experiment was conducted in accordance with the relevant guidelines and regulations, and all participants signed informed consent documents upon commencing the protocol.

#### Materials

**Figure 1:**
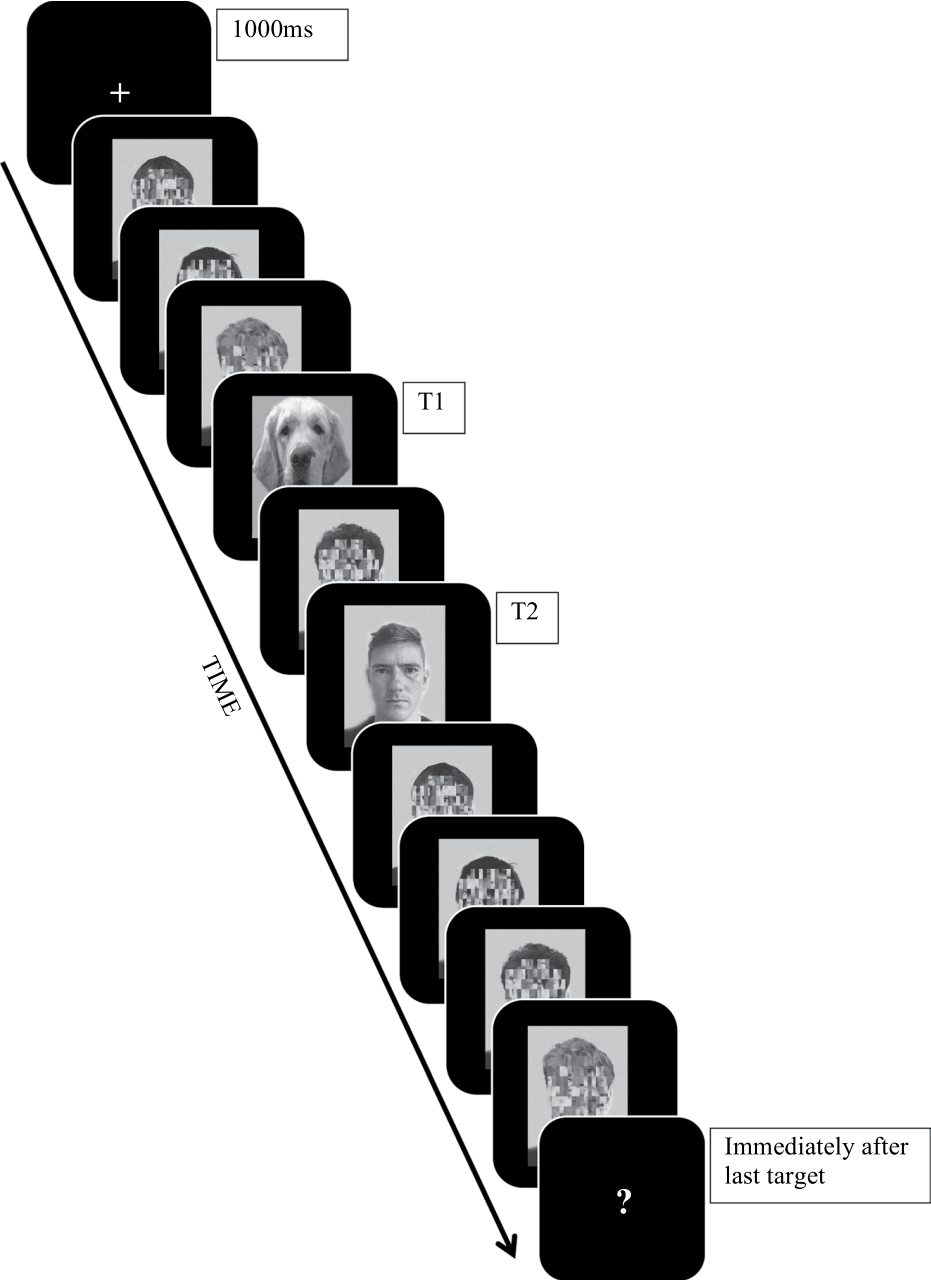
Schematic of the RSVP (lag 2 condition). Each stimuli is presented for 10ms with an inter-stimulus interval of 80ms. The schematic is not to scale – stimuli are disproportionally enlarged to show detail. The figure depicts examples of the scrambled, T1 and T2 stimuli.

Four target faces selected from the Karolinska Directed Emotional Faces Database (KDEF)^60^ were used as second targets in an AB task. Each face was male with a forward facing neutral expression^61,62^ to avoid gender and expression bias. The faces varied in dominance based on the Australiasian first impression ratings of the KDEF database (measured using a seven-point first impression rating scale, see^25,26,63^, available at https://tlab.princeton.edu/databases). On the basis of the ratings, two high dominance faces (mean rating 5.06/7) and two low dominance faces (mean rating 3.85/7) were chosen. The faces were converted to grey scale, reduced in size (width 250 pixels & height 350 pixels), and edited to ensure all faces had similar lighting, head sizes, and eyes at the same level. T1 stimuli consisted of two generic dog faces (drawn from)^58^. The choice of these stimuli was based on the fact that they yielded a robust AB in previous studies^58,64^, as well as because they were visually distinct from both T2 and distractors. Distractor stimuli consisted of twelve KDEF faces that had scrambled facial features (see Fig. 1), also drawn from^58^. Both distractor stimuli and T1 were edited in the same manner as the target faces (grey scale, similar lighting, head sizes, and similar background).

Four fictional curriculum vitae (CVs) were also created for the experiment. Following Dalmaso *et al*.^2^, the CVs depicting varying levels of academic achievement as a proxy for prestige. The CVs all described young males aged between 21 and 24 years of age (average word count = 93). Two “high prestige” CVs depicted individuals that had succeeded in their chosen academic fields (i.e. medicine or film and television). Two “low prestige” CVs depicted individuals that did not succeed in academia (i.e. university drop out or did not complete high school). The two high prestige CVs were each paired with a high dominance face (high status condition) and the two low prestige CVs were each paired with a low dominance face (low status condition).

E-Prime Psychology Software (E-Prime 2.0, https://www.pstnet.com/eprime.cfm) was used to create the task, present stimuli, and collect responses. Participants were seated approximately ~60 cm away from a 24″ BenQ LCD Monitor (100htz refresh rate).

#### Procedure

Prior to the beginning of the AB task, participants were told that they would read a number of CVs and were asked to memorise details about the depicted individuals (names, faces, ages, dates, and locations) in order to correctly answer questions presented throughout the experiment. Participants then read the CVs on the computer, before completing the AB task.

The sequence of events on a typical trial in the AB task can be seen in Fig. 1. Each trial began with a fixation cross, presented in the centre of the display for 1000 ms (size 18, Courier New font, RGB: 255, 255, 255), followed by an RSVP stream of successive images presented at a stimulus-onset asynchrony of 90 ms intervals (10 ms stimulus, 80 ms blank inter-stimulus interval). Four distractor images (scrambled faces) were presented before T1. T2 was then separated from T1 by one (lag 2), three (lag 4) or seven (lag 8) distractor images. After T2, four distractor images were presented. Following the final distractor image, participants were presented with two successive questions on separate screens. The first question was “Did you see a picture of a dog?” while the second question was “Did you see a picture of an unscrambled face?” Participants had five seconds to respond to each of the questions using one of the two marked keys.

Participants completed two blocks of trials. Each block consisted of 144 trials divided equally amongst high and low status T2 faces, at each of the three lags. To ensure participants were observing the stimuli diligently, there were also three types of ‘catch’ trials in each block: trials with no T1 (No T1), trials with no T2 (No T2), and trials with no T1 or T2 (No T1T2). Each type of catch trial was presented three times per lag within each block for a total of 27 catch trials. Therefore, in total, participants completed 171 trials per block and 342 trials overall.

During the task, participants also had to answer six multi-choice questions (two questions before block one, after block one, and after block two), based on the information contained in the CVs presented prior to the experimental trials. These questions were intended to motivate participants to encode the faces and associated CVs. Questions were presented in pairs – one requiring the participant to pick the correct name or information associated with a CV, and one requiring the participant to pick the correct face associated with a CV (e.g. “When did Ted leave high school?”). Between the two experimental blocks (after the second set of questions), participants were also given a short summary of each CV to refresh their memory.

### Results and discussion

Data from three participants were removed - one because their T1 accuracy scores were more than 2.5 *SD* below the mean, one participant was removed because their conditional T2-accuracy score (given T1 was answered correctly; T2|T1) was more than 2.5 *SD* below the mean, and one participant was removed because their T2 accuracy score on ‘catch’ trials (i.e. No T2 & No T1T2) was more than 2.5 *SD* below the mean. As a result, 26 participants were included in the final analysis (*M* = 18.92, *SD* = 1.92, female = 19, male = 7).

#### Knowledge of CVs

Mean accuracy on the six multi-choice questions was 93.00% (range 66.67–100.00%), suggesting the participants accurately encoded information about the faces and associated CVs.

#### T1 Accuracy

Descriptive statistics for T1 are shown in Table 1. Overall T1 accuracy was 95.66% for the high status condition and 96.87% for the low status condition. A 2 (Status: high vs. low) x 3 (Lag: 2, 4, 8) Repeated Measures Analysis of Variance (ANOVA) on these means yielded no significant main effects or interaction (*p* > 0.080, *η*^2^_partial_ < 0.120).

**Table 1:** T1 accuracy as a function of lag and social status.

	Lag 2 *M (SD)*	Lag 4 *M (SD)*	Lag 8 *M (SD)*
High Dominance – High Prestige	95.23 (4.00)	96.09 (3.80)	95.66 (3.73)
Low Dominance – Low Prestige	96.87 (3.58)	96.87 (3.58)	96.87 (3.58)

#### T2|T1 Accuracy

**Figure 2:**
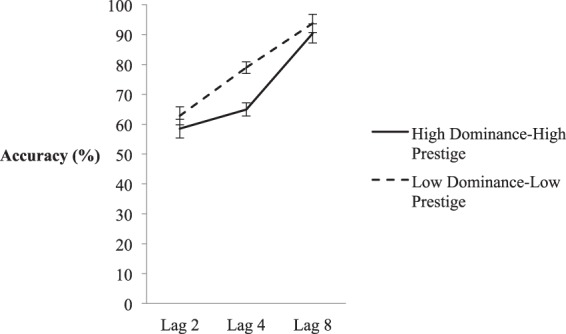
Mean accuracy of the high and low status conditions in Experimental 1a – high prestige CVs paired with high dominance faces, and low prestige CVs paired with low dominance faces. X-axis depicts lags 2, 4, and 8, and within-subjects error bars depict 95% confidence intervals, based on^75^.

Mean second-target accuracy was calculated only on trials in which participants responded correctly to T1 in order to ensure that participants attended to the first target^41^. These mean scores can be seen in Fig. 2. In the following analysis, degrees of freedom were adjusted when needed to compensate for violations of sphericity assumptions (using a Greenhouse-Geisser correction). A 2 (Status) × 3 (Lag) Repeated Measures ANOVA on these means yielded statistically significant main effects of Lag, *F*(1.49, 37.31) = 34.15, *p* < 0.001, *η*^2^_partial_ = 0.577, indicative of a robust AB, and Status, *F*(1, 25) = 46.75, *p* < 0.001, *η*^2^_partial_ = 0.652), as well as a significant Status x Lag interaction effect, *F*(2, 50) = 9.21, *p* = < 0.001, *η*^2^_partial_ = 0.269. Inspection of Fig. 2 suggests this interaction arose because T2 accuracy was higher in the low status condition rather than in the high status condition at Lag 4, at the nadir of the AB. Indeed, performance between these conditions was significantly different – *t*(19) = −6.12, *p* < 0.001, Cohen’s *D* = 0.68. This, in turn, suggests that attention was relatively biased towards low status rather than high status faces.

Unlike results from previous studies that found the focus of reflexive covert attention was biased towards high status individuals^2,22,36^, and contrary to our hypothesis, the results here suggest the opposite pattern when targets compete for attentional resources. As can be seen in Fig. 2, there is an attentional bias towards low status individuals is in the midst of the AB at Lag 4^39–41,57^. However, before addressing the reasons for this difference, we first wished to explore the origins of the attentional bias obtained here in more detail. In particular, it is unclear whether this bias was driven by variations in dominance and/or prestige as these factors co-varied. To address this issue, in Experiment 1b dominance and prestige were placed in opposition: high dominance faces were paired with low prestige CVs, and low dominance faces were paired with high prestige CVs.

## Experiment 1b

### Method

#### Participants

Twenty-nine undergraduate students (*M* = 20.38, *SD* = 4.96, male = 5, female = 24) were recruited from an introductory psychology unit at the University of Western Australia. All participants had normal or corrected-to-normal vision, and received partial course credit for their involvement. None had participated in the previous experiment.

#### Materials and procedure

The stimuli and experimental procedure were identical to Experiment 1a, with the exception that high dominance faces were each paired with a low prestige CV while low dominance faces were each paired with a high prestige CV.

### Results and discussion

Data from two participants were omitted from further analyses because their T2|T1 accuracy was more than 2.5 *SD* below the mean. As a result, 27 participants were included in the final analysis (*M* = 20.48, *SD* = 5.12, female = 23, male = 4).

#### Knowledge of CVs

Mean accuracy on the six multi-choice questions was 89.50% (range 50.00–100.00%), suggesting the participants accurately encoded information about the faces and associated vignettes.

#### T1 Accuracy

Descriptive statistics for T1 are shown in Table 2. Overall T1 accuracy was 93.70% for the high dominance-low prestige condition and 92.90% for the low dominance-high prestige condition. A 2 (Dominance: high dominance-low prestige vs. low dominance-high prestige) x 3 (Lag: 2, 4, 8) Repeated Measures ANOVA yielded no significant main effects or interaction (*p* > 0.080, *η*^2^_partial_ < 0.120).

**Table 2:** T1 accuracy as a function of lag and status.

	Lag 2 *M (SD)*	Lag 4 *M (SD)*	Lag 8 *M (SD)*
High Dominance – Low Prestige	92.90 (7.67)	92.90 (7.67)	92.90 (7.67)
Low Dominance – High Prestige	93.13 (6.14)	94.52 (6.41)	93.44 (6.85)

#### T2|T1 Accuracy

**Figure 3:**
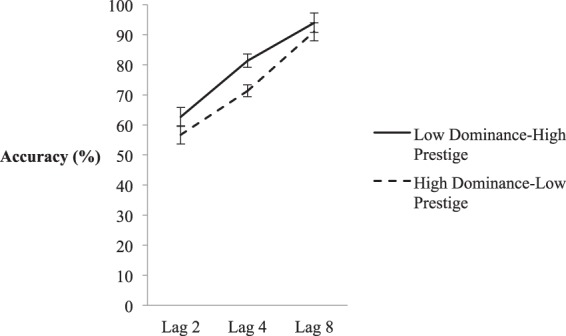
Mean accuracy of the high and low status conditions in Experimental 1b – high prestige CVs paired with low dominance faces. X-axis depicts lags 2, 4, and 8, and within-subjects error bars depict 95% confidence intervals, based on^75^.

Mean T2|T1 accuracy scores were calculated as in Experiment 1a and can be seen in Fig. 3. These scores were analysed with a 2 (Dominance) × 3 (Lag) Repeated Measures ANOVA, which yielded a statistically significant main effect of Lag, *F*(1.50, 36.96) = 63.13, *p* < 0.001, *η*^2^_partial_ = 0.708, indicative of a robust AB, and Dominance, *F*(1, 26) = 16.70, *p* < 0.001, *η*^2^_partial_ = 0.391), as well as a significant interaction, *F*(2, 52) = 5.54, *p* = 0.007, *η*^2^_partial_ = 0.176). As suggested by Fig. 3, this interaction reflects the fact that accuracy was significantly higher in the low dominance-high prestige condition than in the high dominance-low prestige condition at Lag 4, *t*(26) = 4.01, *p* < 0.001, Cohen’s *D* = 0.54, suggesting a reduced AB. In turn, this implies that attention was biased relatively more towards processing of low dominance compared to high dominance faces, despite the fact that high dominance faces were associated with low prestige.

A comparison of results across Experiments 1a and 1b, in which status was consistently associated with dominance but not prestige, replicated the main effects of Lag (*F*(1.51, 74.03) = 83.00, *p* < 0.001, *η*^2^_partial_ = 0.629) and Status (*F*(1, 49) = 47.77, *p* < 0.001, *η*^2^_partial_ = 0.494), as well as a Lag x Status interaction (*F*(2, 98) = 16.80, *p* < 0.001, *η*^2^_partial_ = 0.255). However, neither the main effect of Experiment, nor any interactions involving Experiment were significant (*p* > 0.080, *η*^2^_partial_ < 0.120). This provides additional evidence that it was dominance manipulations (specifically low dominance) rather than prestige manipulations that biased non-spatial attention.

As with Experiment 1a, the findings of Experiment 1b suggest that competition for non-spatial attention is biased towards low status faces – specifically faces associated with low dominance. This outcome contrasts with previous literature, in which eye gaze and reflexive attention were biased towards high status faces^22,36,37^ and influenced by the gaze of high status individuals^2^, in the absence of attentional competition. However it may be premature to assume that prestige manipulations do not affect non-spatial attention. One possible reason for the lack of a prestige effect across the first two experiments may be that attention to facial dominance is driven by a fast, bottom-up processes (occurring after a 10 ms exposure)^24–26^. In contrast, attention to prestige may reflect a slower, top-down process – reflecting time required to link the physical stimulus of the face with previously associated prestige attributes^4,22^.

To test this possibility, a third experiment, Experiment 1c, was conducted to explore the effect of prestige-based status manipulations on non-spatial attention alone, without a concurrent dominance-based status manipulation. The experimental design was similar to Experiment 1a and 1b, except neutral dominance faces (based on)^63^ were used to eliminate the effect of dominance-based status.

## Experiment 1c

### Method

#### Participants

Thirty-five undergraduate students (*M* = 20.47, *SD* = 3.99, 27 female, 8 male) were recruited from an introductory psychology unit at the University of Western Australia. All participants reported normal or corrected-to-normal vision, and received partial course credit for their involvement.

#### Materials and procedure

The prestige manipulations, T1 and distractor stimuli, and experimental procedure were the same as Experiment 1a, with three changes. Firstly, facial stimuli with varying levels of high and low facial dominance were replaced. Instead, four neutral dominance face stimuli were used – based on the Sutherland *et al*.^63^ ratings (mean 4.07/7). They were edited to match the stimuli from Experiment 1a (grey scale, size, lighting, head sizes, and eye level).

Secondly, the second target accuracy question was changed from “Did you see a picture of an unscrambled face?” to “Which person did you see?”. Participants were informed that this is what they would be asked to ensure that they learnt the names with the faces when viewing CVs. Participants were asked to choose one of the four names depicted in the four CVs or a fifth option that said “No face present” (to account for the ‘catch’ trials).

Lastly, participants completed the Perceived Social Status scale (PSS)^65^, which asked them to indicate where they felt individuals depicted in the CVs fit within the broad social hierarchy. Participants were shown a picture of a ladder of ten rungs and asked to imagine the top rung of the ladder as representing the best in society and the bottom rung as representing the worst in society. Participants were then instructed to choose the rung they felt best represented the social standing of the individuals in the CVs. Scores were recorded out of ten, with higher scores indicating a higher social standing.

## Results

Data from three participants were omitted from further analysis. Two participants had accuracy on the ‘catch’ trials’ of more than 2.5 *SD* below the mean, and one participant had a Mahalanobis’ Distance of greater than Chi (6) = 16.81 (*p* = 0.01) for T2 accuracy results. As a result, 32 participants were included in the final analysis (*M* = 20.81, *SD* = 4.01, females = 26, males = 6).

### Perceived social status scale

Results for the 32 participants were averaged across prestige-based status. Mean PSS scores were 7.88 (*SD* = 0.93) and 3.69 (*SD* = 1.22) for individuals depicted in CVs associated with high and low prestige-based status respectively. These means were normally distributed (Skew <|2.00|, Kurtosis <|9.00|). A paired samples *t*-test indicated a statistically significant difference between these PSS means, *t*(31) = 16.25, *p* < 0.001, Cohen’s *D* = 3.90, confirming the effectiveness of our CV-based prestige manipulations.

### Knowledge of CVs

As per the previous experiments, participants were asked six manipulation questions throughout the experiment to ensure they encoded the face stimuli and CVs. The average score for the questions was 5.50 (*SD* = 0.80, minimum = 3). This indicates that participants adequately encoded the face stimuli and CVs.

### T1 accuracy

Descriptive statistics for T1 are shown in Table 3. Overall T1 accuracy was 88.61% for the high prestige condition and 88.15% for the low prestige condition. A 2 (Prestige: high vs. low) x 3 (Lag: 2, 4, 8) Repeated Measures ANOVA on these means yielded no significant main effects or interaction (*p* < 0.600, *η*^2^_partial_ = 0.015).

**Table 3:** T1 accuracy as a function of lag and prestige-based status.

	Lag 2 *M (SD)*	Lag 4 *M (SD)*	Lag 8 *M (SD)*
High Prestige	88.48 (10.46)	89.19 (11.13)	88.15 (11.07)
Low Prestige	88.15 (12.49)	88.15 (12.49)	88.15 (12.49)
*N* = 32			

### T2|T1 accuracy

**Figure 4:**
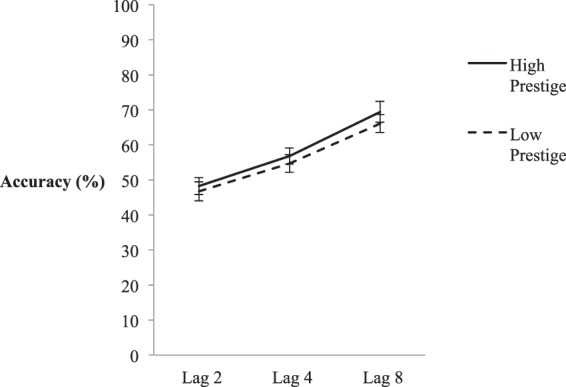
Mean accuracy of the high and low prestige conditions in Experiment 1c. X-axis depicts Lags 2, 4, and 8, and within-subjects error bars depict 95% confidence intervals, based on^75^.

Mean T2|T1 accuracy scores were calculated in the same manner as Experiment 1a and can be seen in Fig. 4. In the following analysis, degrees of freedom were adjusted when needed to compensate for violations of sphericity assumptions (using a Greenhouse-Geisser correction). A 2 (Prestige) x 3 (Lag) Repeated Measures ANOVA yielded a statistically significant effect of Lag – *F*(1.37, 42.46) = 30.55, *p* < 0.001, *η*^2^_partial_ = 0.496, indicative of a robust AB. However the ANOVA did not yield a statistically significant main effect of Prestige – *F*(1, 31) = 0.51, *p* = 0.482, *η*^2^_partial_ = 0.016, nor a significant Prestige x Lag interaction – *F*(2, 62) = 0.22, *p* = 0.806, *η*^2^_partial_ = 0.007. Given a lack of both a significant main effect and an interaction effect, there was no evidence that differences in prestige-based status manipulations affected non-spatial attention.

Experiment 1c showed that manipulations of prestige-based status did not affect non-spatial attention, regardless of whether the manipulations were qualitatively viewed as varying in social status. The results of Experiment 1a-c suggest that non-spatial attention is biased only by low dominance stimuli, contrary to previous social attention and gaze-cueing research^2,22,36,37^.

However, it is important to note that the results thus far simply show that low dominance targets are identified more accurately than high dominance targets. This could mean that attention was biased *towards* low dominance targets, as suggested above, or, logically, that attention was biased *away* from high dominance targets. To disentangle these options, in Experiment 2, we compared performance for high and low dominance targets to neutral dominance targets in order to assess the nature of dominance bias. Given the results of Experiments 1a-c, we expected to find evidence for a bias towards low dominance facial stimuli (i.e. poorer T2 accuracy at Lag 4). Further, given the consistent advantage for low dominance stimuli across Experiments 1a and 1b, we hypothesised that we would obtain evidence for a non-spatial attentional bias towards low dominance faces (i.e. better T2 accuracy for low dominance faces than neutral faces with no difference between T2 accuracy for high dominance and neutral faces at Lag 4). Finally, given prestige did not influence performance in Experiments 1a – 1c, prestige manipulations were not included as variables in Experiment 2.

## Experiment 2

### Method

#### Participants

Forty-three participants (*M* = 19.58, *SD* = 3.02, female = 30, male = 13) were recruited from an introductory psychology unit at the University of Western Australia. All participants had normal or corrected-to-normal vision, and received partial course credit for their involvement. None had participated in the previous experiments.

#### Materials & procedure

The task had four methodological changes from Experiment 1a. First, two additional face stimuli were chosen for the neutral dominance condition from the KDEF database (mean rating 4.07/7). These stimuli were edited in the same manner as Experiment 1a. Second, to accommodate the addition of neutral dominance targets, each block was modified to consist of 42 trials with high, low, and neutral dominance faces (14 of each) presented equally often at each of the three lags. Third, the T2 accuracy question was changed to “Which face did you see?”. Participants were asked to choose a high, low, or neutral dominance face or a fourth option labelled “No face present”. Finally, because prestige was not manipulated in this experiment, CVs and manipulation check questions were omitted.

### Results and discussion

Data from eight participants were removed from the analysis. Four participants had T1 accuracy was more than 2.5 *SD* below the mean. Another four participants had catch trial accuracy more than 2.5 *SD* below the mean. As a result, 35 participants were included in the final analysis (*M* = 19.34, *SD* = 2.60, female = 23, male = 12).

#### T1 Accuracy

Descriptive statistics for T1 are shown in Table 4. Overall T1 accuracy was 92.89% in the high dominance condition, 92.91% in the low dominance condition, and 94.17% in the neutral dominance condition. A 3 (Dominance: high vs. low vs. neutral) x 3 (Lag: 2, 4, 8) Repeated Measures ANOVA yielded no significant main effects or interaction (*p* > 0.050, *η*^2^_partial_ < 0.120).

**Table 4:** T1 accuracy as a function of lag and dominance.

	Lag 2 *M (SD)*	Lag 4 *M (SD)*	Lag 8 *M (SD)*
High Dominance	91.99 (6.87)	93.16 (6.46)	93.52 (7.79)
Neutral Dominance	94.86 (5.50)	93.51 (6.81)	94.15 (5.53)
Low Dominance	91.78 (6.05)	93.96 (5.13)	93.00 (6.70)

#### T2|T1 Accuracy

**Figure 5:**
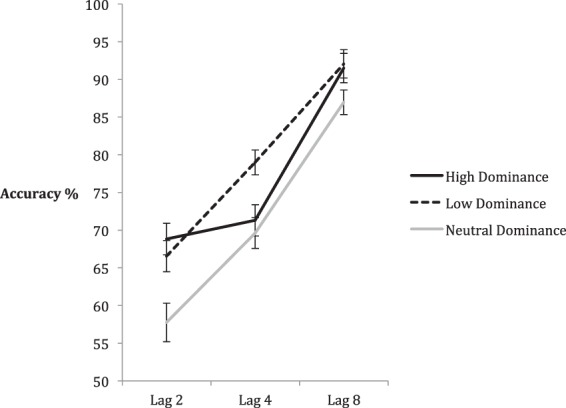
Mean accuracy of the high, low, and neutral dominance conditions across lags 2, 4, and 8. Within-subjects error bars depict 95% confidence intervals, based on^75^.

Mean accuracy was calculated in the same manner as in previous studies and can be seen in Fig. 5. A 3 (Dominance) × 3 (Lag) Repeated Measures ANOVA yielded significant main effects of Lag, *F*(2, 68) = 71.91, *p* < 0.001, *η*^2^_partial_ = 0.679, indicative of a robust AB, and Dominance, *F*(2, 68) = 11.03, *p* < 0.001, *η*^2^_partial_ = 0.245, as well as a significant Dominance x Lag interaction: *F*(4, 136) = 3.86, *p* = 0.005, *η*^2^_partial_ = 0.102.

We focused our initial follow-up comparison on Lag 4, which showed the largest impact of dominance on performance in both previous experiments. A one-way ANOVA comparing performance across dominance levels yielded a significant main effect – *F*(2, 68) = 7.20, *p* = 0.001, *η*^2^_partial_ = 0.175. Fisher’s LSD post-hoc analyses showed significant accuracy differences between the low and high dominance faces (*p* = 0.006, Cohen’s *D* = 0.44) and the low and neutral dominance conditions (*p* = 0.001, Cohen’s *D* = 0.53) but no difference between the high and neutral dominance faces (*p*. = 0.530, Cohen’s *D* = 0.09). This pattern indicates that attention was biased towards low dominance faces rather than away from high dominance ones, thus likely explaining the results from earlier experiments.

Given the graphical evidence from Fig. 5, which suggests that both high and low dominance faces were identified more accurately than neutral dominance faces, we elected to conduct follow-up analyses at Lag 2 and 8 as well. A one-way ANOVA at Lag 2 yielded a significant main effect, *F*(2, 68) = 8.63, *p* < 0.001, *η*^2^_partial_ = 0.202. Fisher’s LSD post-hoc analyses showed significant accuracy differences between high and neutral dominance faces (*p* = 0.001, Cohen’s *D* = 0.53) and between low and neutral dominance faces (*p* = 0.004, Cohen’s *D* = 0.44). However, there was no difference between low and high dominance faces (*p* = 0.401, Cohen’s *D* = 0.12). There was a similar pattern of results at Lag 8. A one-way ANOVA yielded a main effect, *F*(2, 68) = 4.56, *p* = 0.014, *η*^2^_partial_ = 0.118. Fisher’s LSD post-hoc analyses showed significant accuracy differences between high and neutral dominance faces (*p* = 0.030, Cohen’s *D* = 0.40) and between low and neutral dominance faces (*p* = 0.009, Cohen’s *D* = 0.50). However, there was no difference between high and low dominance faces (*p* = 0.745, Cohen’s *D* = 0.06).

In sum, comparison between low and high dominance faces yielded the same pattern of results across lags as observed in the previous experiments. There was a clear advantage for the low dominance faces at Lag 4 in the midst of the AB, as seen in Experiments 1a and 1b – therefore supporting the first hypothesis. However, with the addition of neutral dominance faces in the present experiment, two new insights emerged. First, and confirming the second hypothesis, it seems that the accuracy advantage for low dominance faces at Lag 4 stems from an attentional bias towards these faces, rather than a bias away from high dominance faces (as indicated by similar levels of performance between high and neutral dominance faces). Second, and unexpectedly, both low and high dominance faces were identified more accurately than neutral faces at other lags. This suggests that a second factor is at play with faces that deviate from average dominance. Faces that reflect either low or high dominance may have a general perceptual advantage that leads to more efficient identification, compared to neutral dominance. This perceptual advantage is distinct from the low dominance bias during the height of attentional competition, observed at Lag 4.

## General Discussion

The two primary processes within the dual model of social hierarchy – dominance and prestige – are known to impact how people allocate attention to others, with consistent findings that eye gaze is preferentially focused on high-status individuals^22^ and reflexive attention is biased towards high-status^2,36^. The present research probed whether social status would exert a similar influence on competition for processing resources, such as during the AB.

Experiment 1a demonstrated a significant accuracy advantage for low status faces (both low dominance and low prestige) when used as T2 in the AB, contrary to the original proposed hypothesis. Experiment 1b extended these findings by demonstrating the accuracy advantage occurred for low dominance faces even when they were associated with high prestige, suggesting that dominance rather than prestige was responsible for the performance improvement. In Experiment 1c, it was confirmed that prestige manipulations did not bias non-spatial attention, and therefore the effects seen in Experiment 1a and 1b were due to a low dominance bias. In Experiment 2, we found evidence that the T2 accuracy advantage for low dominance faces seemed to reflect a bias towards attending to low dominance faces rather than a bias against attending to high dominance faces. Importantly, across the experiments, the evidence for an attentional bias towards low dominance targets contrasted with results from previous studies that showed covert attention and eye gaze is reliably biased towards targets high in dominance or prestige^2,22,37^.

One explanation for our results is suggested by the outcome of a previous study that observed a perceptual advantage for low dominance faces. Stewart *et al*.^66^ used the Continuous Flash Suppression (CFS) paradigm to measure the speed in which faces varying from low to high dominance^25^ overcame dynamic noise patterns. Due to their motivational salience and the evolutionary vigilance hypothesis^67^, they expected an advantage for high dominance faces. In contrast, low dominance faces overcame suppression significantly faster than high dominance faces. One explanation for both Stewart’s results and our own findings might be related to resource competition. That is, stimuli associated with varying levels of dominance might differ in their ability to compete for attention with other perceptual inputs. As noted in the Introduction, many models of the AB focus on the importance of inter-item competition^46–49,68^. This framework suggests that T2 stimuli that are resistant to the AB, such as words^69^ or faces^68^, will break through the attentional competition posed by T1 in working memory^70^. With respect to the current study, a similar mechanism may be at work: features specific to low dominance faces may break through the competition posed by T1 significantly more often than both high- and neutral- dominance faces. Similarly, in the Stewart *et al*. study, low dominance faces may have broken through the competition posed by the flash suppression significantly faster than high dominance faces.

Another possibility is that the attentional biases seen here are linked to the relationship between participants’ expectations about how male and female faces should look and the associated allocation of attention. Sutherland *et al*.^71^ found cross-stereotypical faces (e.g. female faces with masculine features) were evaluated more negatively than stereotypical faces (e.g. male faces with masculine features). Moreover, some counter-stereotypical stimuli have been found to preferentially attract spatial attention and prime conscious perception^72,73^. The stereotypical trait dimension for male faces is one of dominance^74^. Thus, the low dominance stimuli may have warranted proportionally greater attention, given the less masculinised male faces differ from the typical male stereotype, compared to high and neutral dominance. Of course, on the basis of the data presented here, these explanations are merely speculative. However, these explanations make distinctly different and testable predictions that are fruitful directions for future research.

One potential limitation of the study was the use of education as a prestige manipulation in Experiments 1a and 1b. Although education-based prestige manipulations were used because they have been shown to modulate gaze cueing^2^, we cannot rule out the possibility that educational achievements were not perceived by our participants as an indicator of social status. In light of this possibility, it will be important for future experiments to directly assess the perceived prestige arising from education-based prestige manipulations, as well as to consider possible alternative sources of prestige such as social media presence.

In conclusion, using the AB paradigm, the current study demonstrated evidence for an attentional bias towards low dominance faces. This effect occurred irrespective of prestige manipulations and seems to be due to an attention bias towards processing low dominance faces rather than a bias against processing high dominance faces. Given the key role of dominance, it is likely that these results reflect the workings of comparatively earlier processing stages tuned to physical features associated with dominance. The findings impact the current theories of social status across social, evolutionary, and cognitive psychology. However additional work is needed to confirm these explanations and to further examine the effects of prestige on non-spatial attentional biases.
